# Poststroke anxiety and depression: epidemiology, mechanisms, and management strategies

**DOI:** 10.3389/fpsyt.2026.1756085

**Published:** 2026-05-04

**Authors:** Yulong Xie, Dandan Wang, Qing Shi, Liping Zhou, Tairong Ai

**Affiliations:** 1 Department of Rehabilitation Therapy, Beilun Dagang Rehabilitation Hospital, Ningbo, Zhejiang, China; 2 Department of Rehabilitation Medicine, The Fourth People’s Hospital of Kunshan, Kunshan, Jiangsu, China; 3 Department of Clinical Psychology, The Third People’s Hospital of Tianshui (Tianshui Psychiatric Hospital), Tianshui, Gansu, China

**Keywords:** emotional health, mechanisms, poststroke anxiety, poststroke depression, rehabilitation, stroke

## Abstract

Poststroke anxiety and depression (PSAD/PSAnx) are common and heterogeneous emotional disorders among stroke survivors, significantly impacting functional recovery, quality of life, and long term prognosis. Their pathogenesis is complex and multifactorial, involving neurobiological alterations, cognitive dysfunction, behavioral responses, and social environmental factors. Cognitive impairments, functional limitations, and insufficient social support can interact to amplify negative emotional responses, forming a vicious cycle of emotional disorders. Pharmacological interventions remain the cornerstone for moderate to severe cases, with selective serotonin reuptake inhibitors (SSRIs) and serotonin norepinephrine reuptake inhibitors (SNRIs) shown to improve mood, cognitive function, and some pain symptoms; individualized dosing and monitoring for adverse effects are essential. Nonpharmacological interventions, including structured rehabilitation, exercise, psychological therapies, and social support, can synergistically enhance neuroplasticity, promote emotional well-being, and improve adherence to rehabilitation. Current evidence is limited by heterogeneous assessment tools, short follow-up periods, and insufficient individualized interventions. Future research should focus on high quality randomized controlled trials, mechanism based stratified management, multimodal interventions, and digital or remote rehabilitation strategies to optimize emotional and functional recovery in stroke survivors. In conclusion, comprehensive interventions targeting PSAD/PSAnx should integrate biological, psychological, and social environmental factors, providing a scientific basis and clinical guidance for improving emotional health, functional restoration, and quality of life.

## 1 Introduction

Stroke is one of the leading causes of mortality and disability worldwide. Its complex pathophysiological processes not only lead to motor, sensory, and cognitive impairments but also significantly affect emotional health. Recent studies have shown that anxiety and depression are the most common and clinically significant psychiatric comorbidities after stroke, collectively referred to as poststroke mood disorders, PSMDs. According to the Global Burden of Disease study, with the continuously increasing number of stroke survivors, early identification and intervention for poststroke mood disorders have become particularly important (1, 2). Systematic reviews indicate that approximately one third of patients develop depressive symptoms at various stages after stroke, while the prevalence of anxiety is approximately 20 to 30 percent, and comorbidity between the two is frequent (3–5). These emotional disorders not only affect psychological experience but are also closely associated with delayed functional recovery, decreased activities of daily living, increased rehospitalization, and elevated mortality risk (6, 7). This evidence underscores the clinical importance of timely recognition and management of poststroke mood disorders as part of routine stroke rehabilitation. Therefore, they have become one of the core factors influencing rehabilitation progress and long term prognosis.

The pathogenesis of poststroke mood disorders involves multifactorial and multilayered interactions. Neurobiological factors are considered primary drivers, including neurotransmitter imbalances such as impaired serotonin, norepinephrine, and dopamine systems, disruption of thalamocortical circuits, and structural damage in key brain regions. In particular, lesions in the frontal lobe, basal ganglia, and limbic system are closely associated with depression (8). In addition, stroke induced neuroinflammatory responses, immune activation, and elevated levels of proinflammatory cytokines such as interleukin six and tumor necrosis factor alpha are also strongly related to emotional disorders (8). Recent studies emphasize the critical role of brain derived neurotrophic factor, with reduced levels negatively correlated with the severity of mood disorders (9, 10). Meanwhile, dysfunction of the hypothalamic pituitary adrenal axis also plays an important role in poststroke anxiety and depression, manifested as abnormal cortisol levels and disrupted circadian rhythms (11, 12).

In addition to biological mechanisms, psychological and social factors play key roles in poststroke mood disorders. Stroke leads to changes in lifestyle, functional dependency, and loss of social roles, which, together with cognitive impairments, reduce patients’ psychological adaptability. Insufficient social support, increased economic burden, and caregiver stress are also closely associated with emotional disorders (13). Complex interactions exist between cognitive deficits and emotional symptoms. Some patients experience impairments in executive function, memory, and attention after stroke, which further exacerbate emotional problems and create a vicious cycle.

Despite the high prevalence and significant impact of poststroke anxiety and depression, clinical recognition and intervention remain insufficient. Suicidality is an important safety concern in this context, as poststroke depression can be accompanied by suicidal ideation and, in some cases, suicidal behavior. This supports explicit assessment of suicidal ideation during mood screening, with appropriate safety evaluation and clear referral pathways for urgent mental health care when risk is identified. Many patients are not systematically screened during the acute and subacute rehabilitation phases, leading to underestimation or delayed diagnosis of emotional disorders. Current interventions include pharmacotherapy, psychological therapies, rehabilitation training, and neuromodulation. Selective serotonin reuptake inhibitors are widely used for depression management, although their efficacy for functional recovery remains controversial (14, 15). Cognitive behavioral therapy has been shown to be safe and effective but is less accessible in stroke populations. Exercise and rehabilitation training can indirectly improve emotional outcomes by promoting neuroplasticity, suppressing inflammation, and modulating emotion related neural networks. Neuromodulation techniques such as transcranial magnetic stimulation and transcranial direct current stimulation have recently shown potential advantages in the management of poststroke mood disorders.

In conclusion, poststroke anxiety and depression are important factors affecting rehabilitation outcomes, and their mechanisms involve interactions across neurobiological, psychological, and social domains. Systematically reviewing their epidemiological characteristics, mechanistic basis, and current management strategies can facilitate early identification, guide individualized interventions, and improve overall rehabilitation outcomes. This review aims to summarize the existing evidence and provide a reference for future research. Although post-stroke depression and anxiety have been discussed in prior reviews, the literature remains fragmented across epidemiology, neurobiology, and rehabilitation practice. The present review adds value by (1) integrating biological, psychological behavioral, and social contextual mechanisms into a rehabilitation-oriented framework that links mechanisms to actionable management choices; (2) distinguishing phase-specific priorities from the acute/subacute period to the chronic stage; and (3) summarizing practical clinical considerations for screening, treatment selection, and monitoring in stroke populations with common constraints such as cognitive impairment, aphasia, frailty, and polypharmacy. Further highlight implementation barriers for neuromodulation and propose a pragmatic, multimodal care pathway to facilitate translation into routine stroke rehabilitation.

## 2 Methods

A comprehensive literature search was conducted in PubMed, Embase, Web of Science, and Google Scholar to identify studies published between January 2000 and October 2025. The search combined terms related to stroke and mood symptoms, including stroke, poststroke, depression, anxiety, and mood disorder, with terms related to management and rehabilitation, including SSRI, SNRI, antidepressant, psychotherapy, cognitive behavioral therapy, exercise, rehabilitation, rTMS, transcranial magnetic stimulation, tDCS, and neuromodulation. Systematic reviews and meta-analyses, randomized controlled trials, and major guidelines or consensus statements were prioritized; key observational studies were also considered when they provided stroke-specific data on risk factors, safety, or implementation. Single case reports and studies not focused on poststroke populations were excluded. Evidence was synthesized thematically across epidemiology, mechanisms, and management strategies, with attention to stage of recovery and common clinical constraints such as cognitive impairment, aphasia, frailty, and polypharmacy.

## 3 Epidemiological characteristics of poststroke anxiety and depression

Poststroke anxiety and depression, PSAD, are the most common psychiatric comorbidities among stroke survivors, characterized by high prevalence, distinct temporal patterns, and individual variability, and they exert significant impact on the rehabilitation process and healthcare systems. Recent meta-analyses indicate that the prevalence of poststroke depression, PSD, varies depending on the assessment method: approximately 24 percent based on clinical interviews and around 29 percent when assessed using standardized scales (16). The onset of PSD exhibits a clear temporal dependence, with most patients developing symptoms during the acute phase (within one month poststroke), peaking in the recovery phase (one to six months), and persisting beyond six months in some cases, with long term prevalence estimated at 10 to 30 percent (16).

The incidence of poststroke anxiety, PSA, is also substantial, measured at 18.7 percent through clinical interviews and 24.2 percent via scale based assessment (17). Anxiety and depression frequently co-occur, with reported comorbidity rates ranging from 20 to 50 percent (18, 19), indicating that these conditions often accompany each other poststroke. Early identification is clinically important and can reduce the risk of chronic emotional disorders. Differences in prevalence across countries and regions may be influenced by healthcare resources, cultural factors, and social support systems, while demographic characteristics are significant predictors. Female sex, older age, lower education level, and a history of psychiatric disorders are associated with a markedly increased risk of PSAD (20). Lesion location and characteristics play a critical role in the development of PSAD. Damage to emotion regulating brain regions, including the left prefrontal cortex, dorsolateral prefrontal cortex, basal ganglia, and thalamus, is strongly associated with PSD and PSA (21, 22). Neuroimaging studies indicate that patients with larger lesion volumes or multiple infarcts have significantly higher depression risk. Additionally, stroke severity, cognitive impairment, motor disability, and speech disturbances are independent risk factors, as these functional impairments indirectly exacerbate emotional problems by reducing daily living ability and social participation (23).

Psychosocial factors are equally important in the development of PSAD. A prior psychiatric history, insufficient family or social support, increased economic burden, maladaptive role transitions, and negative coping strategies are all closely linked to PSD and PSA, with lack of social support particularly associated with increased risk of chronic depression (24, 25). Furthermore, sleep disturbances, chronic pain, medication side effects, and common comorbidities such as hypertension, diabetes, and cardiovascular disease affect neuroplasticity and emotion regulation networks through both physiological and psychological mechanisms, thereby increasing the likelihood of emotional disorders. Regarding clinical outcomes, poststroke depression and/or anxiety are strongly associated with reduced activities of daily living, delayed rehabilitation, and significantly lower quality of life, while also prolonging hospital stay, increasing readmission rates, and elevating overall mortality (26). This evidence highlights the necessity of early identification and comprehensive intervention for stroke patients.

In summary, the occurrence of PSAD involves multidimensional risk factors, including neuropathological features (lesion location, volume, severity), individual characteristics (age, sex, prior psychiatric history), cognitive and functional impairments, and psychosocial environment (social support, economic stress, role adaptation). Understanding these epidemiological characteristics provides a theoretical basis for early screening, risk stratification, and individualized interventions, and aids in developing preventive strategies for high-risk populations.

## 4 Pathophysiological mechanisms of poststroke anxiety and depression

The pathophysiology of poststroke anxiety and depression, PSAD, is complex and involves multidimensional interactions among neurobiological, psychological, behavioral, and social environmental factors, with the central mechanism being structural and functional imbalance induced by brain injury.

### 4.1 Neurobiological mechanisms

Stroke lesions can directly disrupt key emotion regulation networks, including the prefrontal limbic circuit, thalamocortical loop, and basal ganglia circuits. Damage to the prefrontal cortex, hippocampus, amygdala, anterior cingulate cortex, and thalamus, or to the connecting white matter tracts, can lead to impaired structural integrity and functional connectivity of emotion regulation networks (27–29). Neuroimaging studies show that patients with PSAD often exhibit decreased prefrontal activity, reflecting deficits in executive function and inhibitory control, increased amygdala activity, reflecting heightened negative emotional responses, and abnormal functional connectivity between the default mode network and prefrontal control networks (30, 31). These findings support the network disconnection functional imbalance model, in which structural disruption and functional mismatch drive poststroke mood disorders.

The monoaminergic neurotransmitter systems, including serotonin, 5-HT, norepinephrine, NE, and dopamine, DA, play central roles in emotion, motivation, and stress response. After stroke, monoaminergic projection nuclei in the midbrain, such as the locus coeruleus and raphe nuclei, can be impaired. Inflammatory and metabolic abnormalities further suppress monoamine synthesis and release, resulting in decreased levels of 5-HT, NE, and DA in the prefrontal cortex and striatum (32, 33). Monoamine imbalance partially explains poststroke depressive symptoms, including low mood, anhedonia, and anxiety like manifestations. Inflammatory responses are a key driver of neurobiological mechanisms after stroke. Necrotic cells release damage associated molecular patterns, DAMPs, which activate microglia and astrocytes, inducing the release of proinflammatory cytokines such as interleukin-1 beta, IL-1β, interleukin-6, IL-6, and tumor necrosis factor alpha, TNF-α (34, 35). These inflammatory mediators not only exacerbate local neural injury but can also affect distant emotion regulation regions through blood brain barrier permeability changes or paracrine signaling, altering neurotransmitter synthesis and receptor expression (36). Additionally, reperfusion injury and hemorrhage following stroke generate reactive oxygen species, ROS, causing oxidative stress that damages hippocampal and prefrontal neurons and suppresses brain derived neurotrophic factor, BDNF, expression, thereby impairing synaptic plasticity and functional recovery (37). Inflammation, oxidative stress, and BDNF reduction form a self-reinforcing vicious cycle that promotes the persistence of PSAD.

Neuroplasticity, including synaptic plasticity, axonal remodeling, and neurogenesis, is critical for functional recovery and reconstruction of emotion regulation after stroke. Inflammation, oxidative stress, and monoamine imbalance inhibit BDNF and downstream signaling pathways, resulting in impaired synaptic plasticity (38, 39). Clinical and animal studies show that serum BDNF levels are reduced in patients with PSAD and correlate with depression severity and recovery status (40, 41). Functional magnetic resonance imaging studies further indicate abnormal connectivity between the default mode network, central executive network, and salience network, leading to excessive self-referential rumination, heightened sensitivity to negative stimuli, and impaired cognitive control. The interaction of these factors exacerbates depressive and anxious symptoms (42).

**Figure 1:**
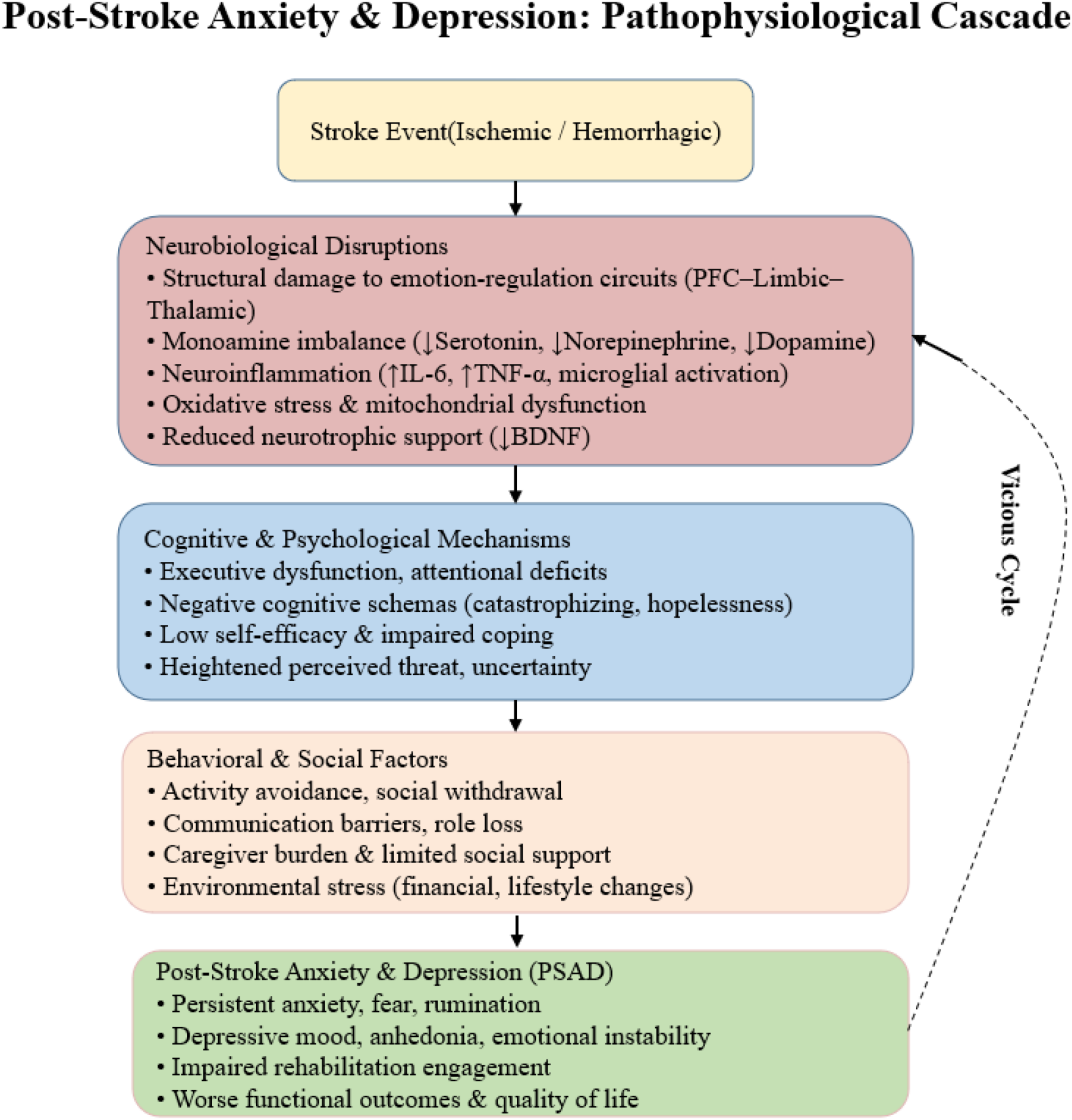
Poststroke anxiety and depression, pathophysiological cascade.

In summary, stroke disrupts emotion regulation networks through direct structural injury and indirect biochemical and inflammatory pathways, dysregulates monoamine and BDNF signaling, induces oxidative stress, and impairs neuroplasticity. Understanding these mechanisms provides a theoretical foundation for mechanism based interventions, including anti-inflammatory therapies, neuroplasticity promoting rehabilitation, monoaminergic drugs, and neuromodulation techniques. The proposed pathophysiological cascade of poststroke anxiety and depression is summarized in **Figure 1**.

### 4.2 Psychological and behavioral mechanisms

Cognitive impairment is a common long term consequence in stroke survivors, primarily affecting domains such as attention, executive function, information processing speed, and memory. This type of cognitive dysfunction reduces patients ability to understand their illness and the rehabilitation process, lowering their participation and adherence to therapy. According to Becks cognitive theory, depression and anxiety reflect automatic negative cognitive processing patterns, such as catastrophizing, low self-efficacy, and pessimistic expectations for the future. Stroke survivors often perceive functional impairment as permanent disability. This negative cognitive bias is not only associated with the severity of poststroke mood disorders but can also predict their long term persistence (43, 44). Cognitive deficits further limit patients ability to comprehend treatment information and make decisions, enhancing feelings of helplessness and anticipated failure, thereby reinforcing negative emotional responses.

Physical dysfunction, such as hemiplegia, speech and swallowing difficulties, and sensory abnormalities, along with decreased activities of daily living, constitutes a significant psychological stressor. These functional limitations not only reduce independence but also disrupt social roles and occupational identity, generating psychological loss, shame, and reduced self-worth, all closely related to poststroke depression. Studies show that the degree of impairment in daily activities is positively correlated with depressive symptoms, whereas reduced social participation significantly increases anxiety risk. In the early rehabilitation phase, uncertainty regarding physical recovery and the risk of chronicity can exacerbate persistent worry and fear, contributing to the maintenance of anxiety symptoms (45).

There is a bidirectional relationship between emotional disorders and behavioral changes. Anxiety and depression can lead to behavioral avoidance, social withdrawal, and reduced activity engagement, while behavioral inhibition further worsens depression by decreasing positive reinforcement, which is a key mechanism of behavioral activation theory. Avoidant behavior also limits participation in rehabilitation, hindering functional recovery and creating a vicious cycle of functional dependence and emotional deterioration (46). Some patients reduce outdoor activities due to fear of falling, recurrent stroke, or social embarrassment. Depressed patients often decrease exercise participation due to lack of motivation. Low activity levels and poor emotional recovery demonstrate a bidirectional causal relationship (47). Additionally, excessive protection or inappropriate care by family members may reinforce passive behavioral patterns, aggravating helplessness and dependency, thereby impeding psychological rehabilitation and functional recovery.

### 4.3 Social and environmental mechanisms

Social and environmental factors play a key role in the development and maintenance of poststroke anxiety and depression. Insufficient social support is one of the most consistent predictors of poststroke mood disorders. Due to limited mobility, communication difficulties, and reduced motivation for rehabilitation, patients experience significantly decreased social interaction. Patients lacking social support often exhibit higher levels of loneliness, persistent emotional distress, and psychological vulnerability (48, 49). Increased caregiver burden further exacerbates psychological stress. High caregiving pressure often leads to reduced self-efficacy, lower adherence to rehabilitation, and enhanced psychological stress responses, thereby significantly increasing the risk of emotional disorders (50).

Additionally, loss of social roles after stroke, including limited occupational function, reduced family role engagement, or decreased social participation, weakens patients sense of identity and constitutes an important psychosocial trigger for anxiety and depression (51). External life events and environmental stressors also significantly affect emotional status. Increased economic burden, changes in family structure, or adjustments in lifestyle are all significantly associated with the occurrence of poststroke anxiety and depression, and patients with lower socioeconomic status are more prone to persistent or severe emotional disorders (52). Overall, social and environmental factors interact with neurobiological and psychological behavioral mechanisms by reducing social support, increasing psychological load, and reinforcing social isolation, collectively influencing the trajectory of poststroke emotional recovery and long term outcomes.

## 5 Management strategies for poststroke anxiety and depression

**Figure 2:**
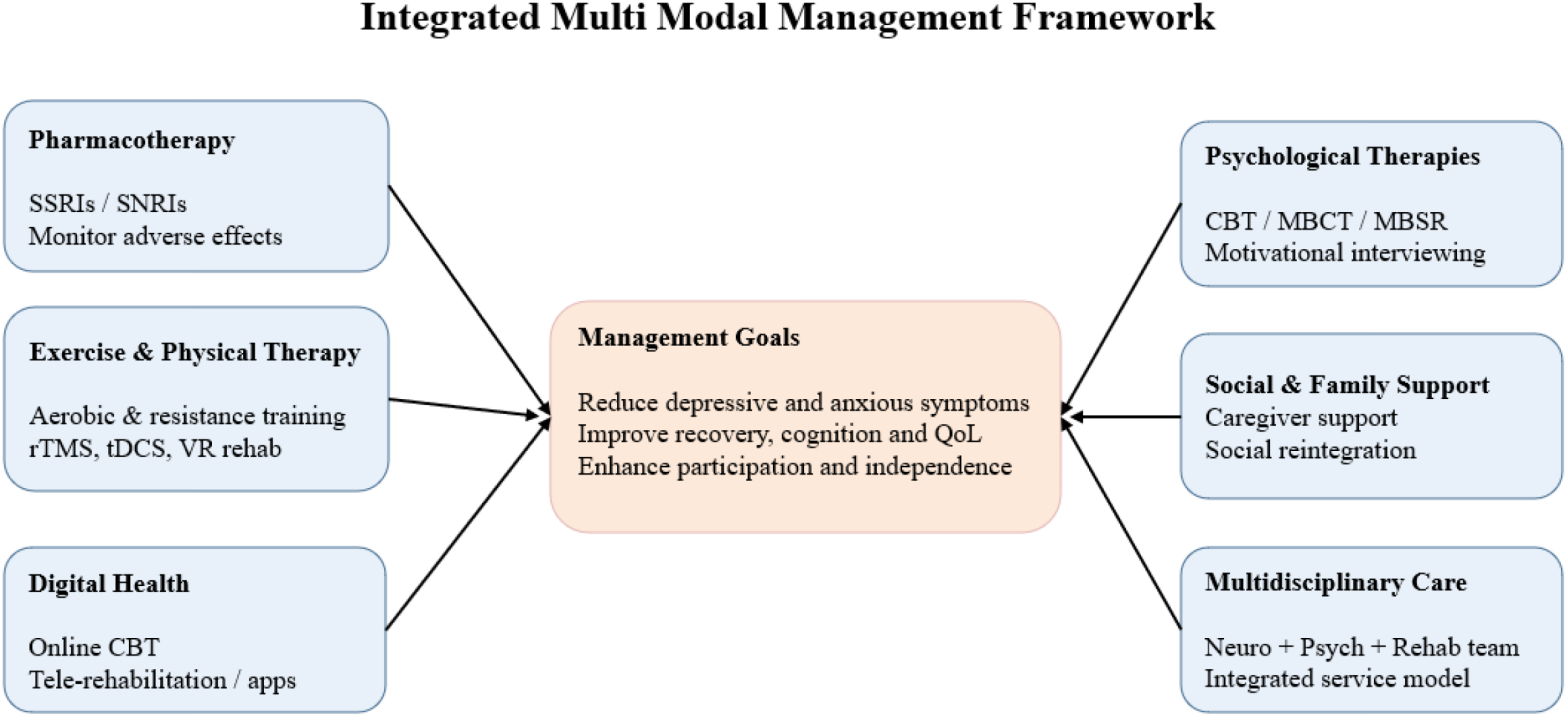
Integrated multi modal management framework.

Management of poststroke mood disorders should follow multimodal, individualized, and evidence based principles, integrating pharmacological treatment, psychological interventions, exercise and rehabilitation training, as well as social and family support. These multidimensional approaches aim to improve emotional symptoms, promote functional recovery, and enhance quality of life. An integrated multimodal management framework for poststroke anxiety and depression is presented in **Figure 2**.

### 5.1 Screening and diagnosis

Screening for poststroke mood disorders should be embedded in routine stroke services because depression and anxiety may be under-recognized and can directly affect rehabilitation participation and outcomes. A structured inventory is recommended for routine screening, followed by a diagnostic interview to confirm the disorder, determine severity, review differential diagnoses, and assess suicide risk (25). The optimal screening timing is not established; however, recommendations support screening during inpatient rehabilitation or early supported discharge, with periodic reassessment during follow-up, particularly at transition points and when there is reduced engagement, sleep disturbance, or declining functional participation (53). For depression, PHQ-2 can be used as a brief first step, with PHQ-9 used for severity grading and monitoring; evidence syntheses and stroke-specific validation studies support the PHQ-9 as a pragmatic option with acceptable psychometric performance in stroke settings (54, 55). Alternative depression scales include CES-D and clinician-rated HDRS, which have also demonstrated utility in stroke cohorts. For anxiety, HADS-A is commonly applied in stroke research and services, and can be used in conjunction with depression measures when both symptom domains are clinically relevant (25).

### 5.2 Pharmacological interventions

Pharmacotherapy is commonly indicated for clinically significant poststroke depression and anxiety, particularly when symptoms are persistent, functionally impairing, or accompanied by suicidal ideation, severe sleep disturbance, or marked rehabilitation disengagement. In stroke populations, treatment selection should be guided by symptom profile, stage of recovery, comorbidities, and concurrent antithrombotic therapy. Conservative initiation and slower titration are advisable in frail older adults and in patients exposed to polypharmacy, with structured monitoring for adverse events (56).

Selective serotonin reuptake inhibitors are commonly used as first-line agents because of established efficacy in depression and anxiety and relatively favorable tolerability. However, high-quality syntheses and large pragmatic trials do not support routine SSRI prescribing for the purpose of enhancing global stroke recovery. A Cochrane review identified 76 trials with 13,029 participants and found no improvement in disability or independence with SSRIs, although a reduction in depression was observed (53). Trials of fluoxetine 20 mg daily for 6 months after acute stroke similarly showed no functional benefit while reducing incident depression, with increased adverse events including fractures, falls, seizures, and hyponatremia (15). These findings support targeted treatment for clinically diagnosed mood symptoms rather than prophylactic prescribing in the early poststroke period.

Stroke-specific safety considerations are central to SSRI prescribing. Potential risks include bleeding when combined with antiplatelet or anticoagulant therapy (57), hyponatremia and falls particularly in older adults and in those with baseline frailty (25). In patients treated with clopidogrel, CYP2C19-inhibiting SSRIs may be associated with a small reduction in clopidogrel effectiveness; selection of agents with less CYP2C19 inhibition can be considered when clinically appropriate (58). Close monitoring is recommended during early titration in high-risk patients, with attention to falls, cognition and alertness, and serum sodium when indicated.

Serotonin norepinephrine reuptake inhibitors may be considered when anxiety symptoms are prominent or when comorbid pain and fatigue contribute to disability and mood symptoms. Venlafaxine extended release is commonly initiated at 37.5 mg daily and titrated based on response and tolerability, up to 225 mg daily. Duloxetine is often initiated at 30 mg daily for one week before increasing to 60 mg daily (59); higher doses require individualized risk assessment in patients with medical comorbidity (60). Blood pressure effects, discontinuation symptoms, and fall risk should be considered in stroke survivors with cardiovascular disease, frailty, or multiple co-medications.

Tricyclic antidepressants demonstrated efficacy in earlier poststroke depression trials but are less favored in routine stroke care because of anticholinergic burden, orthostatic hypotension, sedation, and cardiac conduction risk. In contemporary practice, TCAs are generally reserved for selected patients without substantial arrhythmia risk or severe cognitive impairment, with ECG assessment and careful monitoring (61). Symptom-targeted prescribing can be clinically useful when first-line options are not tolerated. Mirtazapine may be considered when insomnia, appetite loss, or weight loss are prominent; typical dosing starts at 15 mg nightly and can be increased to 45 mg nightly. Bupropion sustained release may be considered for low energy and reduced motivation but should be avoided or used with particular caution in patients with elevated seizure risk; standard titration begins at 150 mg daily and may increase to 300 mg daily, with dose-related seizure risk (62).

Practical stratification is important after stroke because cognitive impairment, aphasia, frailty, and polypharmacy may affect both feasibility and risk benefit balance. In the acute and subacute period, initiation should prioritize clear clinical indications and avoid routine prophylactic antidepressant use solely for neurorecovery, given the lack of functional benefit and the adverse event signal observed in large fluoxetine trials. In fall-prone patients, agents associated with sedation, orthostatic hypotension, or hyponatremia warrant slower titration and closer monitoring. In patients receiving antiplatelet or anticoagulant therapy, bleeding risk should be considered and interactions reviewed systematically (60). Key pharmacological options, typical dose ranges, stroke-relevant evidence highlights, major adverse effects, and subgroup or interaction considerations are summarized in **Table 1** to support practical treatment selection in routine stroke care.

**Table 1:** Pharmacological options after stroke: dosing, evidence, adverse effects, and stroke-specific considerations.

Drug class	Representative agents	Typical post-stroke dose range	Key clinical evidence	Major adverse effects	Stroke-specific considerations
SSRIs (Selective Serotonin Reuptake Inhibitors)	Fluoxetine; Sertraline; Citalopram; Escitalopram	Fluoxetine 20 mg/day; Sertraline 25–100 mg/day; Citalopram/Escitalopram 10–20 mg/day	FOCUS RCT (2019): Fluoxetine 20 mg/day for 6 months showed no functional improvement but reduced incident depression; increased fracture risk (15). Meta-analyses show SSRIs improve depressive symptoms but not disability outcomes (63).	Fractures; falls; hyponatremia; gastrointestinal disturbance; bleeding risk	Not recommended for routine neurorecovery; appropriate for clinically diagnosed depression; assess bleeding risk with antiplatelet/anticoagulant therapy; monitor sodium in elderly.
SNRIs (Serotonin-Norepinephrine Reuptake Inhibitors)	Venlafaxine XR; Duloxetine	Venlafaxine 37.5–225 mg/day; Duloxetine 30–60 mg/day	Systematic reviews suggest benefit for depressive symptoms; limited large stroke-specific RCT. May benefit comorbid anxiety, pain, or fatigue (64).	Hypertension; nausea; discontinuation syndrome; fall risk	Monitor blood pressure in cardiovascular disease; consider polypharmacy interactions; evidence base weaker than SSRIs.
TCAs (Tricyclic Antidepressants)	Nortriptyline; Amitriptyline	Nortriptyline 25–100 mg/day; Amitriptyline 25–150 mg/day	Early small RCT demonstrated efficacy in post-stroke depression; limited contemporary use due to safety concerns (63).	Anticholinergic effects; orthostatic hypotension; sedation; cardiac conduction delay	Avoid in elderly with arrhythmia or cognitive impairment; ECG monitoring recommended.
NaSSA (Noradrenergic and Specific Serotonergic Antidepressant)	Mirtazapine	15–45 mg/day	Limited stroke-specific RCT evidence; used clinically when insomnia, appetite loss, or weight loss are prominent (65, 66).	Weight gain; sedation; dry mouth	Consider in underweight or insomnia-dominant patients; monitor fall risk.
NDRI (Norepinephrine-Dopamine Reuptake Inhibitor)	Bupropion SR	150–300 mg/day	No large stroke-specific RCTs; evidence extrapolated from general major depressive disorder trials (25).	Dose-related seizure risk; insomnia; agitation	Avoid or use caution in patients with seizure history or elevated seizure risk.

SSRI, selective serotonin reuptake inhibitor; SNRI, serotonin norepinephrine reuptake inhibitor; TCA, tricyclic antidepressant; XR, extended release; SR, sustained release; RCT, randomized controlled trial; PSD, poststroke depression; CYP2C19, cytochrome P450 2C19.

### 5.3 Psychological interventions

Psychological therapy is an important component in the management of poststroke depression and poststroke anxiety, and cognitive behavioral therapy has the strongest evidence base among structured psychotherapies evaluated in stroke settings (67). In stroke rehabilitation, CBT is typically adapted to neurological sequelae that influence participation, including attention and memory deficits, reduced processing speed, fatigue, and executive dysfunction (68). Practical adaptations include shorter and more structured sessions, simplified language, repetition of key skills, the use of written and visual cueing, and a stronger emphasis on concrete skill-based elements such as behavioral activation, graded activity scheduling, and problem solving linked to daily participation goals (69). When aphasia is present, access can be improved by using supported communication strategies and pictorial materials, and by involving a caregiver or communication partner to support comprehension, between-session practice, and transfer of skills to everyday routines (70). Stroke-focused trials and syntheses support CBT-informed approaches for mood symptoms after stroke, while also indicating that outcomes may depend on patient selection and the feasibility of delivering cognitively and communicatively accessible interventions within routine services (71).

Differences in psychological priorities across recovery stages are clinically important. In the acute and subacute period, psychological input is often delivered in parallel with active rehabilitation and goal-setting, with an emphasis on providing accessible information, supporting emotional adjustment, and maintaining engagement in therapy (25). Stage-specific adaptations are frequently required because early post-stroke sequelae such as fatigue, attentional impairment, and communication problems can limit tolerance for cognitively demanding interventions. Accordingly, guideline recommendations emphasize reducing cognitive demands through shorter sessions, planned rests, minimizing distractions, and avoiding treatment when the person is fatigued, alongside communication and information adjustments for aphasia, including adapting written materials and minimizing environmental barriers to communication (72, 73).

In the chronic stage, as medical status stabilizes and longer-term participation goals become more salient, psychological interventions can be integrated with community reintegration and self-management planning. Stroke rehabilitation guidance highlights the shift from impairment-focused rehabilitation toward sustained participation outcomes, including social participation and, when relevant, return-to-work planning within coordinated community transitions (74, 75).

### 5.4 Exercise and physical rehabilitation

Exercise intervention is one of the most effective nonpharmacological treatments. Systematic reviews have shown that aerobic exercise, such as walking training, treadmill training, and cycling, can significantly improve poststroke depression. Moderate intensity exercise is generally considered the most effective and feasible (76). Resistance training not only enhances muscle strength but also improves emotional state and executive function, making it suitable for older stroke patients with frailty. Physical factor therapies, including repetitive transcranial magnetic stimulation, transcranial direct current stimulation, transcutaneous electrical nerve stimulation, and virtual reality rehabilitation, are emerging as innovative approaches for treating emotional disorders. The mechanisms, stimulation protocols, evidence base, efficacy summary, and key limitations of rTMS/TBS and tDCS are summarized in **Table 2**. High frequency repetitive transcranial magnetic stimulation targeting the left dorsolateral prefrontal cortex has been confirmed in systematic reviews to improve treatment resistant depression and poststroke depression (77). A recent meta-analysis included 50 RCT with a total of 3852 stroke patients and systematically evaluated the effects of various noninvasive brain stimulation techniques on post stroke depression and anxiety. The findings indicated that these interventions produced overall beneficial effects on emotional symptoms and also contributed, to some degree, to concurrent improvements in cognitive and motor function. Among the different techniques, transcranial magnetic stimulation demonstrated the most consistent therapeutic advantage. Combining transcranial magnetic stimulation at different stimulation frequencies with working memory training yielded the greatest benefits in alleviating depressive symptoms and enhancing activities of daily living. In addition, the combination of transcranial direct current stimulation with psychological therapy, as well as the integration of transcranial ultrasound with working memory interventions, was associated with more pronounced improvements in post stroke anxiety (78).

**Table 2:** Noninvasive brain stimulation for poststroke anxiety and depression.

Technique	Mechanism	Common targets and stimulation protocols	Stroke-relevant evidence base	Key findings and efficacy summary	Key Limitations and interpretation
rTMS/TBS	Time-varying magnetic fields induce cortical currents and modulate excitability within prefrontal-limbic networks implicated in affect regulation (79–82).	High-frequency rTMS (≥ 5–10 Hz) or intermittent TBS targeting the left DLPFC for depressive symptoms; low-frequency rTMS (≤ 1 Hz) targeting the right DLPFC for anxiety and hyperarousal (82, 83). Typical course: 2–4 weeks, 5 sessions/week.	Stroke-specific systematic reviews and meta-analyses (82, 84); meta-analysis of combination with antidepressants (80); network meta-analyses comparing protocols (85).	Meta-analyses show rTMS significantly reduces HAMD scores versus sham (79, 82, 86). Some studies report improvements in activities of daily living (84). Combined therapy may enhance efficacy (80). Network meta-analyses rank high-frequency left DLPFC protocols among the most effective (87).	High heterogeneity in frequency, intensity, and treatment duration; small-to-moderate sample sizes; variation in stroke stage; limited long-term follow-up; limited anxiety-specific outcomes (79, 87).
tDCS	Low-intensity direct currents modulate cortical membrane potential and network-level plasticity, potentially enhancing prefrontal control of emotion processing (88, 89).	Common montage: anodal stimulation over left DLPFC with cathode over contralateral supraorbital region; intensity 1–2 mA; duration 2–4 weeks; sometimes combined with rehabilitation or psychotherapy (88, 89).	Stroke-specific systematic reviews and meta-analyses (79, 89); network meta-analyses including tDCS protocols (87).	Meta-analyses suggest tDCS reduces depressive symptoms versus sham (79, 89). Some RCTs report functional improvements (90), though overall effect sizes are generally smaller than rTMS in comparative analyses (85). Limited but promising evidence for anxiety reduction.	Substantial protocol variability (montage, intensity, session number); small samples; mixed comparator conditions; optimal dose and long-term efficacy remain uncertain (79, 89).

Several pragmatic factors influence whether noninvasive brain stimulation can be translated into routine stroke care. For rTMS, implementation commonly depends on access to specialized equipment, trained personnel, and standardized safety procedures, including pre-treatment risk assessment and the capability to manage adverse events (91). Treatment courses also require repeated sessions over multiple weeks, creating travel and scheduling burdens that may be challenging for stroke survivors with mobility limitations, fatigue, caregiver dependence, or rural residence. Service-level analyses and health technology assessments further note that workforce capacity, clinic throughput, and reimbursement policies are major determinants of adoption and sustainability (92). For tDCS, the lower equipment burden may improve scalability, but clinical implementation still requires protocol standardization, operator training, and supervision models to ensure safety and adherence. Regulatory and governance considerations remain relevant, including how home-supervised stimulation is monitored and integrated with clinical follow-up. These system-level constraints should be acknowledged when interpreting the evidence base and when proposing care pathways for poststroke mood disorders.

### 5.5 Social and family support

Social support plays a decisive role in psychological recovery after stroke. Stable family support can enhance emotional resilience, reduce the risk of poststroke depression, and improve participation and adherence in rehabilitation (93). Excessive caregiver burden or lack of professional guidance may decrease the patient’s sense of self-worth and indirectly affect rehabilitation progress. Loss of social roles, functional limitations, and reduced social participation are important predictors of poststroke depression, while social reintegration, including community activities, leisure activities, and restoration of occupational roles, is considered a key pathway for improving emotional outcomes (94).

This review has several limitations. Although the literature was identified using a structured search, the work remains a narrative synthesis rather than a fully systematic review, and study selection and emphasis may therefore be subject to selection bias. The included evidence spans heterogeneous study designs, populations, stroke stages, intervention protocols, and outcome measures, which limits direct comparability across studies and reduces confidence in conclusions about relative effectiveness between modalities. In addition, much of the primary evidence in poststroke anxiety and depression is constrained by small sample sizes, variable control conditions, short follow up, and inconsistent reporting of treatment fidelity and adverse events, which may affect reliability and generalizability. Language restrictions and publication bias cannot be excluded.

## 6 Conclusion

Poststroke anxiety and depression are a class of emotional disorders with significant heterogeneity. Their etiology is complex and multidimensionally interactive, involving neurobiological changes, psychological and behavioral responses, and social environmental factors. This review integrates evidence from different dimensions, showing that poststroke mood disorders are not only accompanying issues during stroke recovery but also key factors affecting neuroplasticity, functional reconstruction, and long term prognosis. Therefore, emotional management should be regarded as an important component of the stroke rehabilitation system rather than an ancillary intervention. Current evidence supports the adoption of multimodal and integrative management strategies, including the coordinated application of pharmacological therapy, psychological treatment, rehabilitation training, exercise and physical factor therapy, nursing support, and social resource interventions. Nonpharmacological interventions such as exercise and physical therapy have shown unique advantages in improving mood, promoting neuroplasticity, and enhancing rehabilitation adherence, providing an important supplement to conventional treatment. However, current clinical practice still lacks unified assessment procedures, interdisciplinary collaboration mechanisms, and individualized treatment pathways, resulting in delayed diagnosis and insufficient intervention. Future research needs to further clarify the neural mechanisms of poststroke anxiety and depression, establish precise classification and risk prediction models, and promote the development of assessment tools based on multimodal imaging, biomarkers, and artificial intelligence. In addition, it is necessary to conduct high quality randomized controlled trials to validate the optimal combination of comprehensive interventions and long term efficacy, and to explore the feasibility of digital rehabilitation and remote interventions in real world settings. In summary, the identification and management of poststroke anxiety and depression are currently at a critical stage of transition from single treatment to integrated, precise, and rehabilitation oriented approaches. Mechanism based stratified management, interdisciplinary collaboration models, and innovative rehabilitation technology interventions are expected to significantly improve emotional health, functional recovery, and quality of life in stroke survivors.
