# Supplementary material for: Poststroke anxiety and depression: epidemiology, mechanisms, and management strategies
Source: Front Psychiatry. 2026 May 4;17:1756085. doi: 10.3389/fpsyt.2026.1756085 (PMC13180854; doi:10.3389/fpsyt.2026.1756085)
Supplement: Supplementary file 1 [file Presentation1.pptx]

## Slide 1
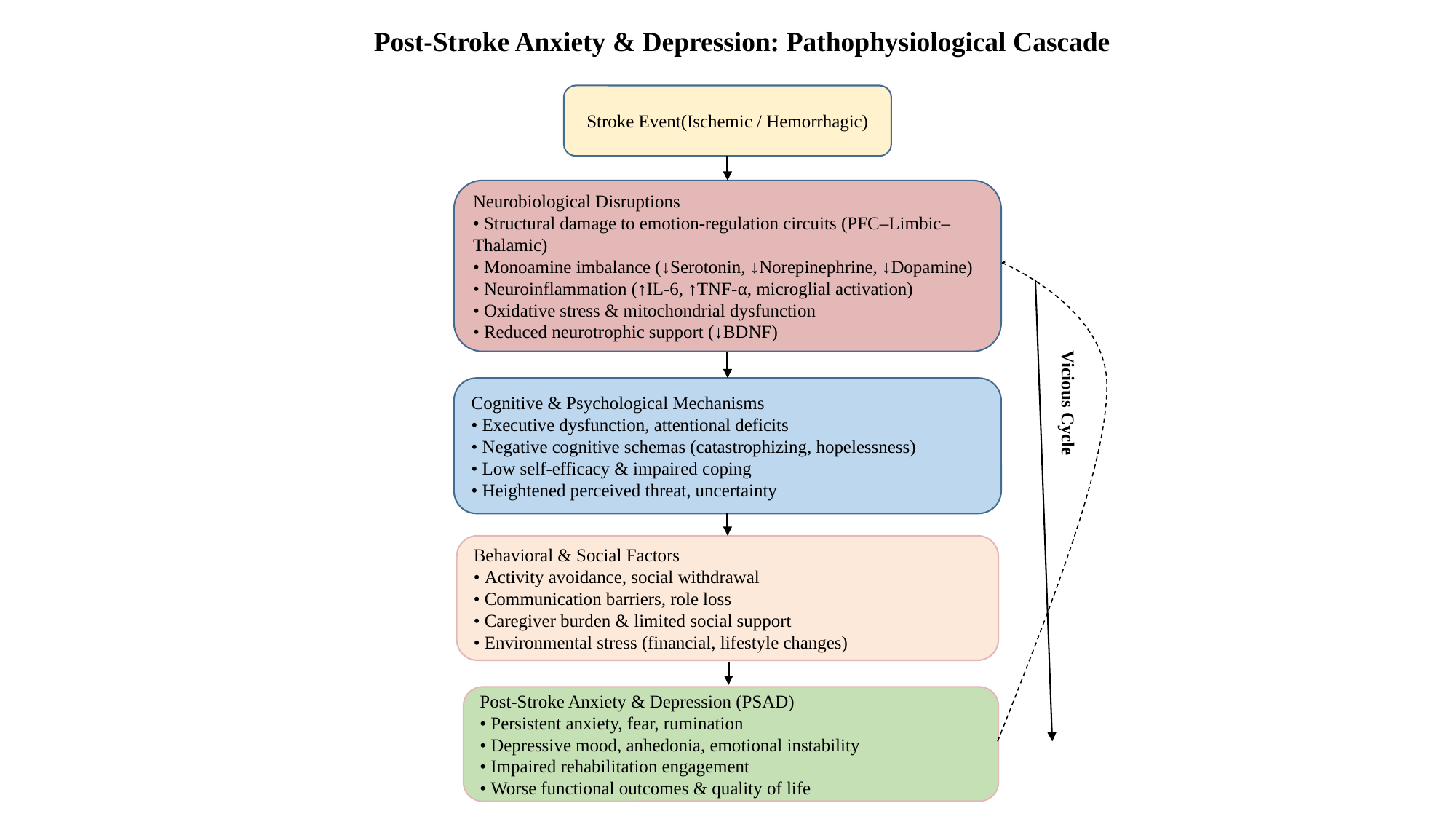

Post-Stroke Anxiety & Depression: Pathophysiological Cascade
Stroke Event(Ischemic / Hemorrhagic)
Neurobiological Disruptions
• Structural damage to emotion-regulation circuits (PFC–Limbic–Thalamic)
• Monoamine imbalance (↓Serotonin, ↓Norepinephrine, ↓Dopamine)
• Neuroinflammation (↑IL-6, ↑TNF-α, microglial activation)
• Oxidative stress & mitochondrial dysfunction
• Reduced neurotrophic support (↓BDNF)
Cognitive & Psychological Mechanisms
• Executive dysfunction, attentional deficits
• Negative cognitive schemas (catastrophizing, hopelessness)
• Low self-efficacy & impaired coping
• Heightened perceived threat, uncertainty
Vicious Cycle
Behavioral & Social Factors
• Activity avoidance, social withdrawal
• Communication barriers, role loss
• Caregiver burden & limited social support
• Environmental stress (financial, lifestyle changes)
Post-Stroke Anxiety & Depression (PSAD)
• Persistent anxiety, fear, rumination
• Depressive mood, anhedonia, emotional instability
• Impaired rehabilitation engagement
• Worse functional outcomes & quality of life

## Slide 2
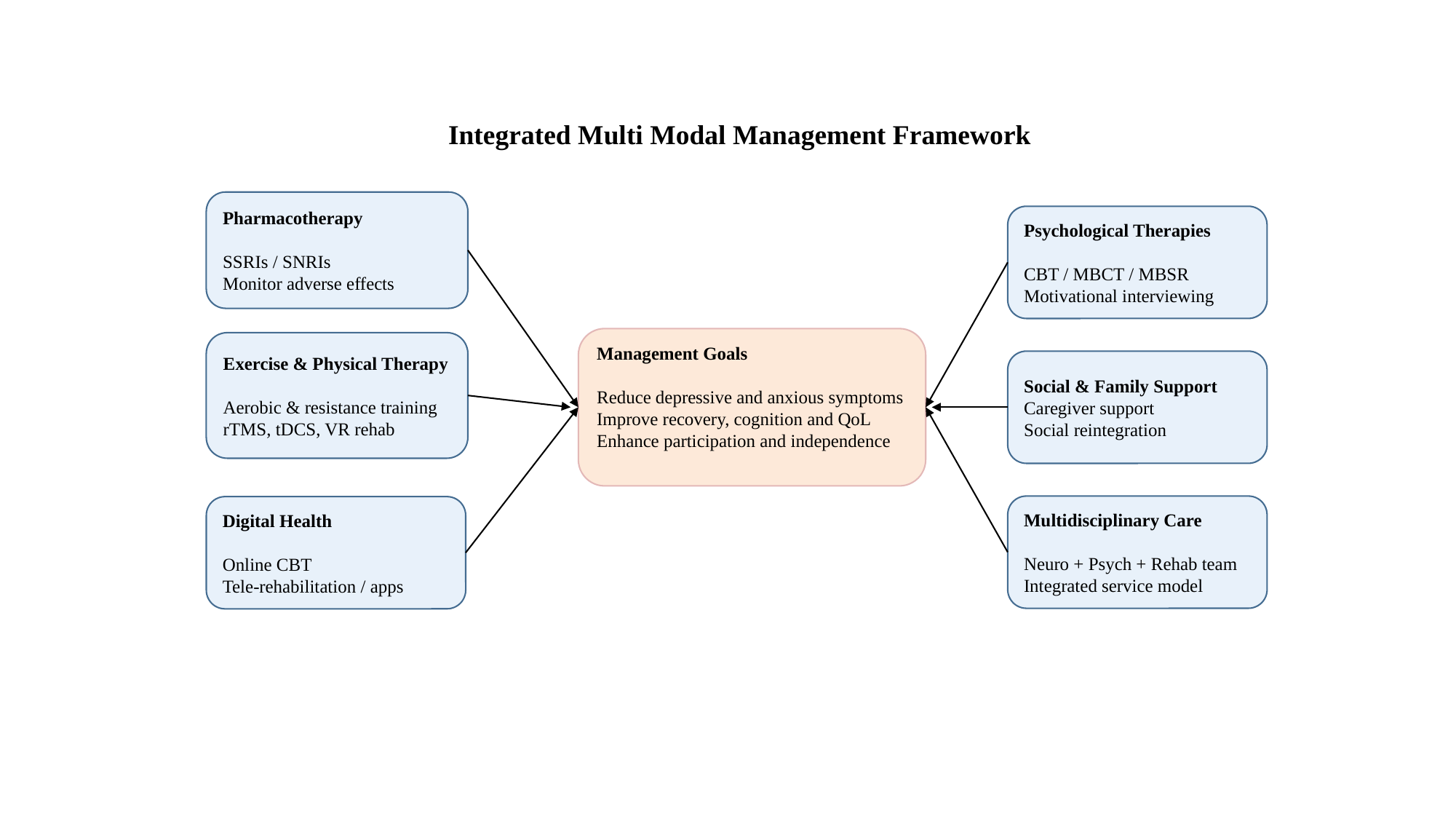

Integrated Multi Modal Management Framework
Pharmacotherapy
SSRIs / SNRIs
Monitor adverse effects
Psychological Therapies
CBT / MBCT / MBSR
Motivational interviewing
Management Goals
Reduce depressive and anxious symptoms
Improve recovery, cognition and QoL
Enhance participation and independence
Exercise & Physical Therapy
Aerobic & resistance training
rTMS, tDCS, VR rehab
Social & Family Support
Caregiver support
Social reintegration
Multidisciplinary Care
Neuro + Psych + Rehab team
Integrated service model
Digital Health
Online CBT
Tele-rehabilitation / apps
